# Intraneural Platelet-Rich Plasma Injections for the Treatment of Radial Nerve Section: A Case Report

**DOI:** 10.3390/jcm7020013

**Published:** 2018-01-29

**Authors:** Unai García de Cortázar, Sabino Padilla, Enrique Lobato, Diego Delgado, Mikel Sánchez

**Affiliations:** 1 Service of Orthopedic Surgery and Traumatology, Basurto Hospital, 48013 Bilbao, Spain; gartxo25@hotmail.com (U.G.d.C.); enrique.lobatomenendez@osakidetza.eus (E.L.); 2 University Institute for Regenerative Medicine and Oral Implantology-UIRMI, University of the Basque Country, Vitoria, 01007 Vitoria-Gasteiz, Spain; sabinopadilla@hotmail.com; 3 Advanced Biological Therapy Unit, Hospital Vithas San José, 01008 Vitoria-Gasteiz, Spain; diego.delgado@ucatrauma.com; 4 Arthroscopic Surgery Unit, Hospital Vithas San José, 01008 Vitoria-Gasteiz, Spain

**Keywords:** platelet-rich plasma, radial nerve section, intraneural injections

## Abstract

The radial nerve is the most frequently injured nerve in the upper extremity. Numerous options in treatment have been described for radial nerve injury, such as neurolysis, nerve grafts, or tendon transfers. Currently, new treatment options are arising, such as platelet-rich plasma (PRP), an autologous product with proved therapeutic effect for various musculoskeletal disorders. We hypothesized that this treatment is a promising alternative for this type of nerve pathology. The patient was a healthy 27-year-old man who suffered a deep and long cut in the distal anterolateral region of the right arm. Forty-eight hours after injury, an end-to-end suture was performed without a microscope. Three months after the surgery, an electromyogram (EMG) showed right radial nerve neurotmesis with no tendency to reinnervation. Four months after the trauma, serial intraneural infiltrations of PRP were conducted using ultrasound guidance. The therapeutic effect was assessed by manual muscle testing and by EMG. Fourteen months after the injury and 11 months after the first PRP injection, functional recovery was achieved. The EMG showed a complete reinnervation of the musculature of the radial nerve dependent. The patient remains satisfied with the result and he is able to practice his profession. Conclusions: PRP infiltrations have the potential to enhance the healing process of radial nerve palsy. This case report demonstrates the therapeutic potential of this technology for traumatic peripheral nerve palsy, as well as the apt utility of US-guided PRP injections.

## 1. Introduction

The radial nerve is the most frequently injured nerve in the upper extremity, especially in patients with multiple injuries [1]. It may be damaged by several mechanisms, such as direct nerve trauma, complex humerus fracture, compression, (i.e., Saturday night palsy), iatrogenic lesions, neuritis or, more rarely, tumors and lead ingestion [2]. Clinically, these patients present a wide range of symptoms from weakness to complete paralysis of the elbow, forearm, wrist, finger, and thumb extension, failure of forearm supination, thumb abduction, and triceps reflex abolition, with or without sensory deficit in the back side of the forearm and in back and radial side of the hand. The loss of active extension of the wrist removes the mechanical advantage to grab things and grip hard.

Numerous options in treatment have been described for radial nerve injury. The approach may depend on the cause of the injury, and observation is often enough in diagnosing most cases, with a spontaneous resolution of the palsy [3]. However, in severe situations, treatment includes primary repair, neurolysis, nerve grafts, or tendon transfers. A few years ago, tendon transfers were the predominant reconstructive option for radial nerve injuries, but this choice is often technically difficult, and the outcomes are not always satisfactory [2,3,4]. In recent years, evidence has been accumulating in both preclinical and clinical settings indicating that platelet-rich plasma (PRP) products, and fibrin scaffolds obtained from this technology hold therapeutic potential as a neuroprotective, neurogenic, and neuroinflammatory modulator system [5,6,7], and an enhancer of sensory and motor functional nerve-muscle unit recovery [8,9,10,11,12]. PRP is a liquid-to-gel fibrin matrix injectable scaffold that, once applied on the injured area, either as a filler, suturable membrane, or scaffold tissue, fibrinolysis breaks the fibrin down, thereby releasing cell signaling molecules such as nerve growth factor (NGF), brain derived neurotrophic factor (BDNF), insulin-like growth factor 1 (IGF-1), platelet derived growth factor (PDGF), vascular endothelial growth factor (VEGF), hepatocyte growth factor (HGF), fibrin, fibronectin and vitronectin.These biomolecules have been shown to be instrumental instructive and permissive agents involved in the control of stem cell-like myelinating Schwann Cells (SC) activation, macrophage polarization, as well as in the active resolution of inflammation, angiogenesis, and fibrogenesis, thereby acting as key drivers of nerve function recovery [5,11]. This manuscript describes the case of a patient who was treated for a traumatic radial nerve section 48 h after injury.

## 2. Case Report

### 2.1. Case Description

**Figure 1:**
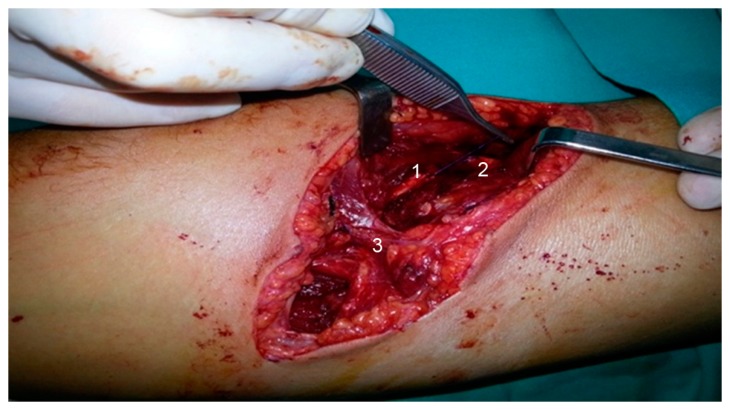
First surgical revision of the wound. Section of the radial nerve, distal tendon and muscle belly of the biceps brachii. **1**: distal end of the radial nerve; **2**: proximal end of the radial nerve; **3**: Lacertus fibrosus.

The study was conducted in accordance with the Declaration of Helsinki, and case report was approved by the Ethics Committee of Hospital Universitario Basurto (CS/UGA/1217, approved date: 21 December 2017). The patient was a healthy 27-year-old man with no history of interest to this report. He was a professional plumber, amateur cyclist, and snowboarder who, during an assault, suffered a deep and long cut (about 10 cm) with a knife in the cubital fossa of the right arm, extending proximally towards the radial region. Immediately after the knife cut, the patient had lost full functional ability to recruit the dependent muscles of the radial nerve, and suffered dysesthesia in the region. Due to the alarming clinical extent of the cut, the patient went to the emergency department of the nearest hospital. Upon arrival, four hours after the injury, the patient was taken to the operating room and under general anesthesia, surgical revision of the wound was conducted. After revision, the entire section of the radial nerve, distal tendon and muscle belly of the biceps brachii, and a section of the sensory branch of the musculocutaneous nerve were observed. No vascular damage was evident in the major vessels of the region. Next, labeling of proximal and distal ends of radial nerve with non-absorbable monofilament suture (polypropylene) was conducted. Moreover, an end-to-end suture of the distal tendon of biceps brachii was performed, which followed a postoperative period without incident, with progressive recovery of strength and mobility. The skin was provisionally closed with metal staples (Figure 1). Finally, the limb was immobilized by means of a dorsal plaster splint. After this emergency surgery, the patient was sent to his referral hospital in order to repair the lesions described previously.

The patient came to our clinic 45 h after injury. The sensory examination showed hypoesthesia in all dependent musculocutaneous nerve areas and hypoesthesia with a feeling of numbness in the radial dependent region. Tinel’s sign was found in the suture zone without progression to the distal nerve. Manual muscle testing (MMT) was 0/5 for all dependent regions of the radial nerve distal to the injury, except for the common extensor muscle of fingers and for 5th finger extensor which was 1/5.

Forty-eight hours after injury the patient underwent general anesthesia, and a second wound review, where the previously-described injuries were observed. A reinforcing of the brachial biceps tendon suture was performed. An end-to-end suture of the radial nerve and of the sensory branch of musculocutaneous nerve was also conducted. The suture was done without a microscope so only an epineural suture was performed with non-absorbable Ethilon 8/0 suture. In addition, coverage of the nerve sutures was associated with vein grafts obtained from superficial veins of ipsilateral palmar wrist region. The skin was closed with metal staples and the member was immobilized with a posterolateral plaster splint.

Ten days after the second-look surgery, the patient reported good pain control without fever or systemic symptoms. Immobilization was removed, and a good evolution of the surgical wound was observed so the staples were removed, and the antibiotic treatment was halted. Physical examination did not reveal variations from before second surgery. The plaster splint was replaced with a thermoplastic splint in extension in order to begin assisted physiotherapy immediately by rehabilitation service. The patient underwent a rehabilitation program that included electrostimulation and specific therapy. This therapy consisted primarily in exercises, like stretching of the fingers and wrist, and motor skills with the hand. Despite the rehabilitation and a comprehensive monitoring program, functional progress was not satisfactory and there were no clinically-significant changes three months after the second surgery. Therefore, an electromyogram (EMG) was required, showing right radial nerve neurotmesis with no tendency to reinnervation.

Given the circumstances, it was decided to apply an intraneural ultrasound (US)-guided injection of PRP. The results observed in animal studies as well as in other similar cases and in prior orthopaedic experience using PRP were key in choosing this treatment [10,13].

### 2.2. Platelet-Rich Plasma Preparation

PRP was elaborated according to PRGF^®^-Endoret^®^ technology (BTI Biotechnology Institute, Vitoria-Gasteiz, Spain). Briefly, a total of 36 ml of peripheral venous blood was withdrawn into 9-mL tubes containing 3.8% (*w*/*v*) sodium citrate. Blood was centrifuged at 580 g for eight minutes at room temperature. The upper volume of plasma, which contains a similar platelet count to that of peripheral blood, was drawn off and discarded in a collection tube. The 2-mL plasma fraction, located just above the sediment red blood cells, but not including the buffy coat, was collected in another tube and carried to the injection room ready for use. This plasma contains a moderate enrichment of platelets (two- to three-fold the platelet count of peripheral blood) with scarce leucocytes, being a P2-x-Bβ PRP according to the classification system proposed by DeLong et al. [14]. To initiate clotting, calcium chloride (10% *w*/*v*) was added to the liquid PRP aliquots just before injection. All procedures were performed under sterile conditions.

### 2.3. US-Guided Injection Technique

**Figure 2:**
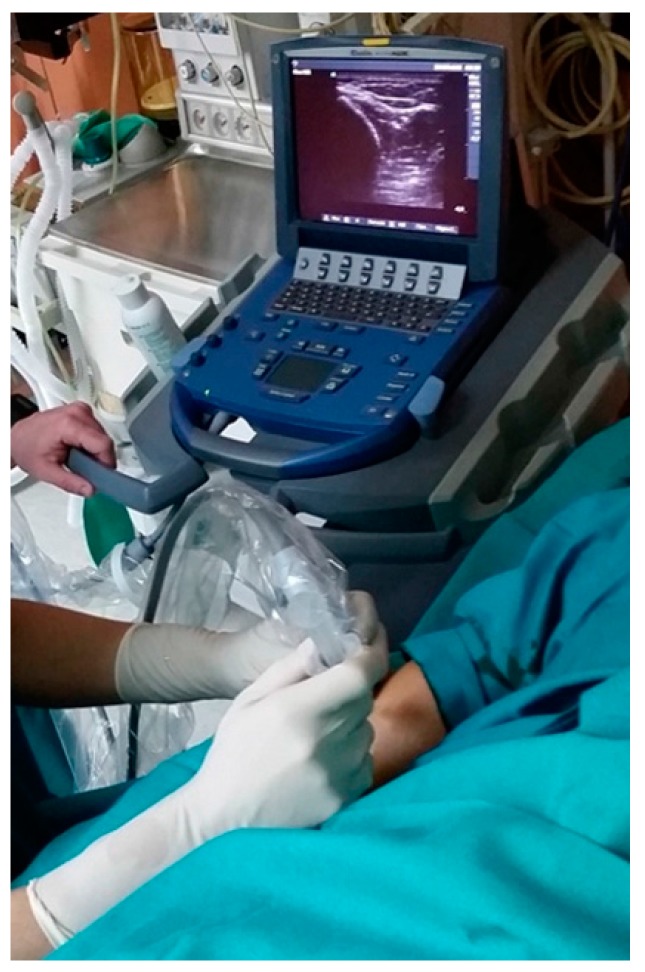
US-guided plasma rich in growth factors injection for the radial nerve. The procedure was performed in sterile conditions in an outpatient setting. The multi-frequency linear probe was aligned with the long axis of the radial nerve, and a 22-gauge needle (25 mm) was inserted into the radial nerve. The accuracy of platelet-rich plasma (PRP) infiltration was confirmed by direct visualization by US imaging.

For each injection, US examination was carried out by an experienced radiologist together with the same orthopedic surgeon. First, the injured site was located and, because it seemed to have continuity, it was decided to perform the injection in the suture area. The injection was performed using a 22-gauge needle just below the probe to allow visualization of the needle and ensure an intraneural infiltration of the autologous preparation. Four milliliters of PRP was injected softly within the epineurium and also around the injured site (Figure 2). In total, five injections were performed, the first three with two-week intervals followed by two more injections two months after the first infiltration. The frequency of these injections rested on the clinical and EMG outcomes.

### 2.4. Clinical Evolution after the First Injection

Three months after the first PRP injection the patient reported subjective clinical improvement in sensitivity and the MMT was 3/5 for all dependent regions of the radial nerve distal to the injury, except for the common extensor muscle of finger in brachiorradialis that was 1/5. Due to these encouraging results new infiltrations were applied.

**Figure 3:**
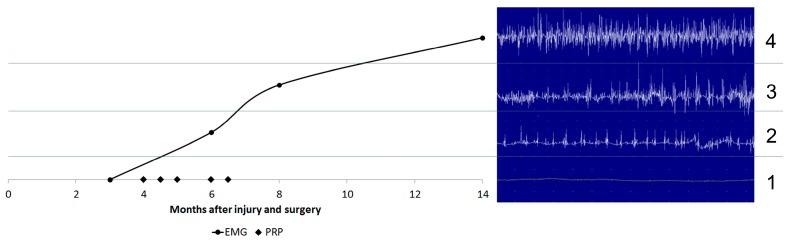
Electromyographic control from surgery up to date. Three months after surgery (**1**); sSix months after surgery, three after first PRP injection (**2**); eight months after surgery, five after the first PRP injection (**3**); and one year after surgery, nine months after first PRP injection (**4**). EMG: electromyogram; PRP: platelet-rich plasma infiltrations.

Five months after the first injection, with a total of five intraneural PRP injections, EMG showed a right radial neuropathy with reinnervation in the muscle dependent on radial nerve below the elbow. At that time, the MMT had improved and presented 5/5 in the common extensor muscle of the finger, carpis radialis longus extensor, carpis radialis brevis extensor, digiti minimi extensor; 4/5 in the indicis extensor; and 2/5 in the brachiorradialis. Sensitivity demonstrated almost full recovery in the radial nerve distribution area. At that point, the patient started a new rehabilitation program that included activities that he performed at work, so he could achieve sufficient functionality to return to his profession. Fourteen months after the injury and 11 months after the first PRP injection, functional recovery was achieved. The EMG showed a complete reinnervation of the musculature dependent of the radial nerve (Figure 3).

## 3. Discussion

In the field of orthopedics and plastic surgery, the process of nerve regeneration and target organ reinnervation is a difficult challenge. Fueled by the drawbacks posed by autologous nerve autografts, several biomedical engineering strategies have recently emerged. These include nerve guidance conduits and scaffolds, which can incorporate and deliver neurotrophic factors and support cells into nerve guidance conduits or fibrin gels, and target organ stimulation through intramuscular injections of growth factors [5,11,15]. Nevertheless, these new strategies are infrequently used either as a main therapeutic approach or as an adjuvant for nerve regeneration. Research studies using PRP in animals and humans are encouraging [6,8,9,10,11,12,16]. Surgical procedures and results for nerve repair and their results are conditioned by several factors, such as the patient´s age, the cause and level of the lesion, associated injuries, duration of denervation, the length of the nerve defect, the type of repair, the use of electrophysiological recordings, and the surgeon´s experience [17]. The radial nerve functional recovery after five perineural and intraneural injections of PRP presented in this case report is consistent with the results published by other teams using PRP as a therapeutic tool to assist nerve repair [13,18,19,20,21,22], although some subtle differences in procedures are notable. In a case study from Kuffler et al., a long ulnar nerve gap 12 cm in length was bridged with autologous PRF as a filler in a collagen tube 3.25 years after an ulnar nerve trauma leading to recovery of both muscle and sensory function in a 58 year old patient [8]. Moreover, in a recent series of cases of surgical nerve repair, Kuffler et al. reported functional recovery in 18 patients under 58 years whose nerve gaps of 2–16 cm were treated with collagen tubes filled with PRP, 0.5–3 years after the traumatic injury [18]. No patients suffered adverse events. Two clinical trials using PRP as an adjuvant therapeutic tool to assist nerve surgery reported protective and clinical beneficial effects. Scala et al. applied PRP gel to the excision site and around facial nerve endings in eight patients with mixed tumors undergoing superficial parotidectomy [20]. In the same direction, Hibner et al. coated the pudendal nerve with PRP after pudendal neurolisis in 10 patients where transgluteal decompression of the pudendal nerve had failed [21]. Sanchez et al. and Malahias et al. applied the liquid dynamic fibrin formulation of PRP as an injectable adjuvant to treat common peroneal nerve palsy and carpal tunnel syndrome respectively, with successful nerve function recovery [13,22].

In the case reported here, the existence of associated nerve injuries has a negative effect in the postoperative recovery because of an increasing amount of scar tissue and extensive muscle involvement [23]. Furthermore, when the reparation is done later than 24 h from injury, it is always necessary to remove at least 1 mm of nerve stump [24]. The suture was performed 48 h after the damage, without microscope or intraoperative electrophysiological recordings. According to an algorithm for radial nerve reconstruction, a tension-free end-to-end repair of the radial nerve section was conducted, but without removing the nerve stump. Only an epineural suture was carried out, without joining the fascicles together. Owing to these procedural conditions, the postoperative evolution was not satisfactory, and three months after the surgery the patient presented no improvement in either sensory or motor function; muscle atrophy was emerging despite physiotherapy. At this point EMG study was requested and a right radial nerve neurotmesis was detected with no tendency to reinnervation. Based on results from the scientific literature and before considering other surgical options, such as neurolysis, nerve grafting, or even tendon transposition, we decided to use the PRP approach to create a favorable environment for nerve repair by injecting perineural and intraneural PRP. Once activated, it becomes a liquid, sponge-like, viscous and malleable dynamic and transient fibrin scaffold that fills the nerve gaps and adheres to ECM structures of the damaged tissue [25]. The suitability of PRP as a therapeutic tool is underpinned by its gradual and sustained release of growth factors and other molecular signals from the autologous platelet-rich plasma derived fibrin matrix, as well as its bioresorbable, biocompatible, versatile, and plastic properties. In contrast to a bolus delivery modality of GFs, which has been shown to be less efficacious in the repair process [26] tissue fibrinolysis mediates in PRP´s gradual and sustained release of several GFs and other biomolecules [27]. At present, 14 months after the injury and 11 months after the first PRP injection, functional recovery has been completed, with the exception of the brachiorradialis function that still presents a grade of three in manual muscle testing. The EMG shows a complete reinnervation in the musculature of the radial nerve dependent, with the exception of an injury focus on the exit branch to the brachiorradialis. The patient is satisfied with the result and he is able to practice his profession. Although this is an excellent result, it is reasonable to expect a spontaneous recovery without using PRP. However, the patient’s clinic and previous experience suggest that he would have presented a motor and sensory deficit, as he had prior to infiltrations [13]. Indeed, a better and faster clinical outcome might have been obtained by applying PRP infiltrations in the early phase, starting in the emergency room following the surgical process shown in the Sánchez et al. work on sheep [10].

The significant clinical outcome of this case report in parallel with the other results described here supports the therapeutic use of PRPs as versatile and safe biological products to be harnessed by surgeons and clinicians as an adjuvant therapeutic tool to enhance the robust intrinsic nerve repair processes, and overcome a post-traumatic and neuropathic inhibitory microenvironment by delivering neurotrophic and neurotropic factors. PRP may assist nerve conduit guidance and grafts as a filler, as a liquid in intraneural and perineural ultrasound-guided injections in nerve entrapments and fibrosis, and as a scaffold to bridge or wrap the injured nerve gap.

## 4. Conclusions

PRP infiltrations have the potential to enhance the healing process of radial nerve palsy. This case report suggests the therapeutic potential of this technology for traumatic peripheral nerve palsy, as well as the apt utility of US-guided PRP injections.
